# Theoretical Design and Synthesis of Caged Compounds Using X‐Ray‐Triggered Azo Bond Cleavage

**DOI:** 10.1002/advs.202306586

**Published:** 2024-01-15

**Authors:** Koki Ogawara, Osamu Inanami, Hideo Takakura, Kenichiro Saita, Kohei Nakajima, Sonu Kumar, Naoya Ieda, Masato Kobayashi, Tetsuya Taketsugu, Mikako Ogawa

**Affiliations:** ^1^ Laboratory of Bioanalysis and Molecular Imaging Graduate School of Pharmaceutical Sciences Hokkaido University Sapporo Hokkaido 060‐0812 Japan; ^2^ Laboratory of Radiation Biology Graduate School of Veterinary Medicine Hokkaido University Sapporo Hokkaido 060‐0818 Japan; ^3^ Quantum Chemistry Lab Department of Chemistry, Faculty of Science Hokkaido University Sapporo Hokkaido 060‐0810 Japan; ^4^ Institute for Chemical Reaction Design and Discovery (WPI‐ICReDD) Hokkaido University Sapporo Hokkaido 001‐0021 Japan

**Keywords:** azo compounds, cancer, cleavage reaction, radical reactions, reaction mechanism

## Abstract

Caged compounds are frequently used in life science research. However, the light used to activate them is commonly absorbed and scattered by biological materials, limiting their use to basic research in cells or small animals. In contrast, hard X‐rays exhibit high bio‐permeability due to the difficulty of interacting with biological molecules. With the main goal of developing X‐ray activatable caged compounds, azo compounds are designed and synthesized with a positive charge and long π‐conjugated system to increase the reaction efficiency with hydrated electrons. The azo bonds in the designed compounds are selectively cleaved by X‐ray, and the fluorescent substance Diethyl Rhodamine is released. Based on the results of experiments and quantum chemical calculations, azo bond cleavage is assumed to occur via a two‐step process: a two‐electron reduction of the azo bond followed by N─N bond cleavage. Cellular experiments also demonstrate that the azo bonds can be cleaved intracellularly. Thus, caged compounds that can be activated by an azo bond cleavage reaction promoted by X‐ray are successfully generated.

## Graphical abstract

Hydrated electrons are highly reactive species produced by X‐ray irradiation. By suitably designing azo compounds, azo bonds can be efficiently cleaved by the hydration electrons generated upon X‐ray irradiation. This cleavage reaction is thought to progress in two steps: an N─N bond cleavage following a two‐electron reduction. Furthermore, X‐ray‐induced azo bond cleavage reactions can also efficiently occur in cells.

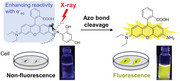

## 1 Introduction

In life science research, caged compounds are frequently used for analyzing biological processes and functions.^[^ ^1^, ^2^, ^3^ ^]^ However, the visible to near‐infrared light commonly used in this technology is absorbed and scattered by biological materials, limiting its use to basic research in cells or small animals. Hard X‐rays (wavelength of 0.001–10 nm) exhibit high bio‐permeability in living organisms, even in humans, due to the weak absorption from biological molecules.^[^ ^4^, ^5^ ^]^ Thus, they can be used in diagnostic imaging techniques such as X‐ray computed tomography to detect deep lesions in the human body.^[^ ^6^ ^]^ Furthermore, hard X‐rays can ionize molecules such as water at high doses, generating radical species that can be used for radiation therapy and radiosensitive nanoparticles enhancing effects of radiation therapy were developed.^[^ ^7^ ^]^ Generally, these radical species immediately react with endogenous scavengers (e.g., amino acids and glutathione) and disappear in the living body.^[^ ^8^, ^9^ ^]^ However, if exogenously administered compounds possess higher reactivity towards these X‐ray‐derived radical species than endogenous scavengers, such compounds might be used as X‐ray activatable caged compounds in living bodies. X‐ray activatable caged compounds can release bioactive substances or drugs deep inside the body where X‐rays are irradiated. Among the radicals generated by X‐rays, hydroxyl radicals and hydrated electrons (e^−^_aq_) account for a large proportion. Their G values, which means the number of generated molecules by the absorption of 100 eV X‐rays, are 2.74 for hydroxyl radicals and 2.63 for hydrated electrons respectively.^[^ ^10^, ^11^, ^12^ ^]^ Whereas hydroxyl radicals possess a high oxidation‐reduction potential (ORP), hydrated electrons have a low ORP. Thus, it was envisaged that oxidation or reduction reactions induced by these two radical species could be applied to X‐ray activatable caged compounds. In previous reports, compounds that can be activated by hydroxyl radicals were used for the oxidation of azidos^[^ ^13^ ^]^ and hydroxylation of phenyl groups.^[^ ^14^ ^]^ In addition, the reduction of *N*‐oxides^[^ ^15^ ^]^ and elimination from quaternary ammonium compounds^[^ ^16^ ^]^ by hydrated electrons have also been used for X‐ray activatable caged compounds. All these studies investigated the reactivity of various candidates to identify the most reactive compounds for hydroxyl radicals and hydrated electrons, however the reaction mechanisms were not elucidated yet. In order to develop X‐ray activatable caged compounds that react efficiently, it is necessary to establish a theoretical design and elucidate the oxidation or reduction mechanisms.

Azo compounds are suitable candidates for X‐ray activatable caged compounds. Previous reports suggested that 1‐phenylazo‐2‐naphthol is decomposed by hydrated electrons generated by kilo‐order Gy of γ‐rays, producing a variety of degradation products. Among these, aniline deriving from the parent materials was detected.^[^ ^17^ ^]^ We hypothesized that X‐ray activatable caged compounds triggered by azo bond cleavage could be developed, although efficiency and selectivity shall be improved for use in caged compounds. In this study, we rationally designed azo compounds that were highly reactive to hydrated electrons and investigated whether the azo bond cleavage was induced by X‐rays in cuvette studies and cell experiments. Furthermore, we aimed to elucidate the reaction mechanism of azo bond cleavage induced by X‐rays through a comprehensive investigation of experiments and quantum chemical calculations. Especially, the vertical electron affinity (VEA) of each compound was evaluated by quantum chemical calculations to investigate the resonant reduction by hydrated electrons.

## 2 Results and Discussion

### 2.1 Design of Azo Compounds Undergoing X‐Ray Induced Azo Bond Cleavage

**Scheme 1:**
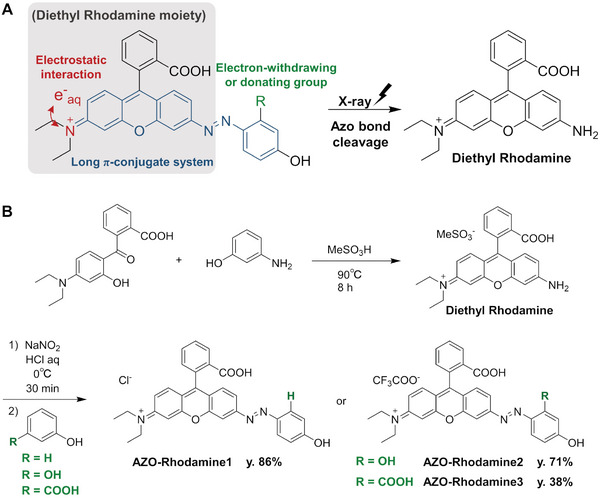
X‐ray activatable compounds, AZO‐Rhodamine1‐3. A) Molecular design of AZO‐Rhodamine1‐3 and their X‐ray‐promoted reactions. A rhodamine moiety bearing a cation and long π‐conjugate system was introduced in the azo structure aiming at enhancing reactivity with hydrated electrons. B) Synthetic scheme for the preparation of AZO‐Rhodamine1‐3.

e^−^_aq_ possess a monovalent negative charge and high reducing potential. Thus, compounds with a positive charge and long π‐conjugated systems can be highly reactive with hydrated electrons due to electrostatic interactions as well as their lower lowest unoccupied molecular orbital (LUMO) energy (**Scheme** **1**A). We decided to modify the amino group of Diethyl Rhodamine, which features a positive charge and long π‐conjugated system, to an azo group. Thus, we designed and synthesized AZO‐Rhodamine1 carrying a phenylazo group by azo coupling of Diethyl Rhodamine and phenol (Scheme 1B). In addition, to investigate the effect of functional groups at the benzene moiety of the azo group, hydroxy and carboxy groups were introduced at the ortho position relative to the azo bond (AZO‐Rhodamine2 and AZO‐Rhodamine3, respectively). AZO‐Rhodamine2 and AZO‐Rhodamine3 were synthesized by azo coupling between Diethyl Rhodamine and resorcinol and *m*‐hydroxybenzoic acid, respectively. The structures of all these compounds were confirmed by nuclear magnetic resonance and mass spectroscopy analyses.

### 2.2 Azo Bond Cleavage Induced by X‐Ray Irradiation

**Figure 1:**
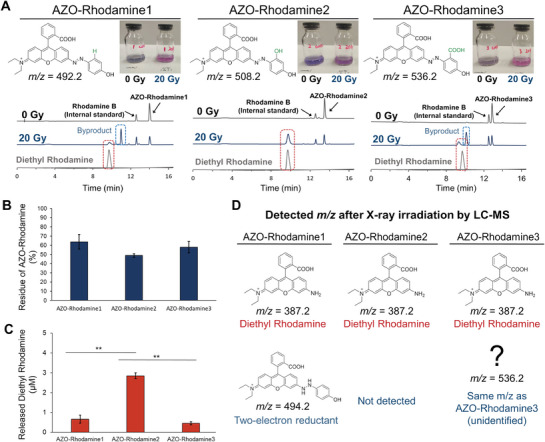
HPLC analysis of AZO‐Rhodamine1‐3. A) HPLC charts of AZO compounds after X‐ray irradiation at 0 and 20 Gy. Photographs of the solutions after/before irradiation are also shown above the HPLC chart. The HPLC charts show that all three compounds reacted upon X‐ray irradiation, resulting in the formation of Diethyl Rhodamine. In the case of AZO‐Rhodamine1 and 3, other peaks were observed. B) and C) Quantitative analysis of remaining AZO‐Rhodamine1‐3 and released Diethyl Rhodamine after irradiation. The error bars represent the SEM (AZO‐Rhodamine1 of B): n = 5, AZO‐Rhodamine1 of C), 2 and 3: n = 3, Tukey‐Kramer test, ^**^P < 0.01).

Firstly, we examined whether the azo bond of AZO‐Rhodamine1‐3 could be cleaved and Diethyl Rhodamine could be released upon X‐ray irradiation using a linear accelerator. Owing to the low solubility of AZO‐Rhodamine1‐3, X‐ray irradiation was performed under conditions involving 40% MeOH. The G‐value of solvated electrons produced from MeOH was reported to be 1.1 as measured by microsecond radiolysis.^[^ ^18^ ^]^ Therefore, the G‐values of hydrated electrons under the condition of phosphate buffer containing 40% MeOH can be considered to be lower or close to 2.63, which corresponds to those observed under the condition of 100% water. Each 5 µM solution of AZO‐Rhodamine1‐3 in phosphate buffer containing 40% MeOH was bubbled with argon to remove oxygen, which acts as a radical quencher, and then irradiated with 20 Gy X‐ray. In this condition, the amount of hydrated electrons were calculated to be 5.4 µm based on the G‐value. Since hydroxyl radicals are quenched by MeOH, the reactivity of hydrated electrons was investigated under these conditions. The irradiated solutions were analyzed via HPLC using Rhodamine B as an internal standard. After X‐ray irradiation, the peaks of AZO‐Rhodamine1‐3 decreased and new peaks with the same retention time as Diethyl Rhodamine appeared (**Figure** **1**A). In the case of AZO‐Rhodamine1 and AZO‐Rhodamine3, additional peaks were observed. The residual percentage of each azo compound was 64, 49, and 58% for AZO‐Rhodamine1, AZO‐Rhdamine2, and AZO‐Rhodamine3, respectively (Figure 1B). On the other hand, the concentration of released Diethyl Rhodamine from each azo compound was 0.66, 2.8, and 0.45 µm for AZO‐Rhodamine1, AZO‐Rhodamine2, and AZO‐Rhodamine3, respectively (Figure 1C). These results suggest that the yields of released Diethyl Rhodamine were significantly different among AZO‐Rhodamine1‐3 despite similar reactivity to hydrated electrons.

Next, X‐ray‐irradiated solutions were analyzed by LC‐MS. As a result, the same mass‐to‐charge ratio as Diethyl Rhodamine (*m/z* = 378.2) was found in each solution of AZO‐Rhodamine1‐3. In addition, a mass‐to‐charge ratio of byproducts was found in the solutions of AZO‐Rhodamine1 and 3. The mass‐to‐charge ratio of byproduct in the case of AZO‐Rhodamine1 (*m/z* = 494.2) was greater than that of AZO‐Rhodamine1 by +2 (Figure 1D). The mass‐to‐charge ratio of byproduct of AZO‐Rhodamine3 (*m/z* = 536.2) was the same as AZO‐Rhodamine3, but the peak had a different retention time. So far, no structure has been identified.

**Figure 2:**
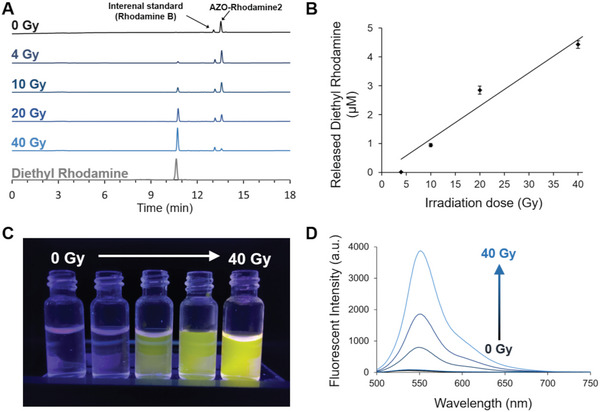
X‐ray dose dependency of AZO‐Rhodamine2. A) HPLC chart of AZO‐Rhodamine2 after irradiation at 0, 4, 10, 20, and 40 Gy. B) Quantitative analysis of Diethyl Rhodamine released from AZO‐Rhodamine2 (5 µM) with Rhodamine B as an internal standard. Error bars represent the SEM (n = 3). C) Fluorescence of an AZO‐Rhodamine2 solution excited by a UV lamp. D) Fluorescence spectra of X‐ray irradiated solutions.

We investigated the X‐ray dose dependency of AZO‐Rhodamine2. The amount of Diethyl Rhodamine was quantified after X‐ray irradiation at 4, 10, 20, and 40 Gy and Diethyl Rhodamine linearly increased against the X‐ray dose (**Figure** **2**A,B). Moreover, the fluorescence of Diethyl Rhodamine increased (Figure 2C,D).

### 2.3 Quantum Chemical Calculations for the Azo Cleavage Reaction Between Hydrated Electrons and AZO‐Rhodamine1‐3

**Figure 3:**
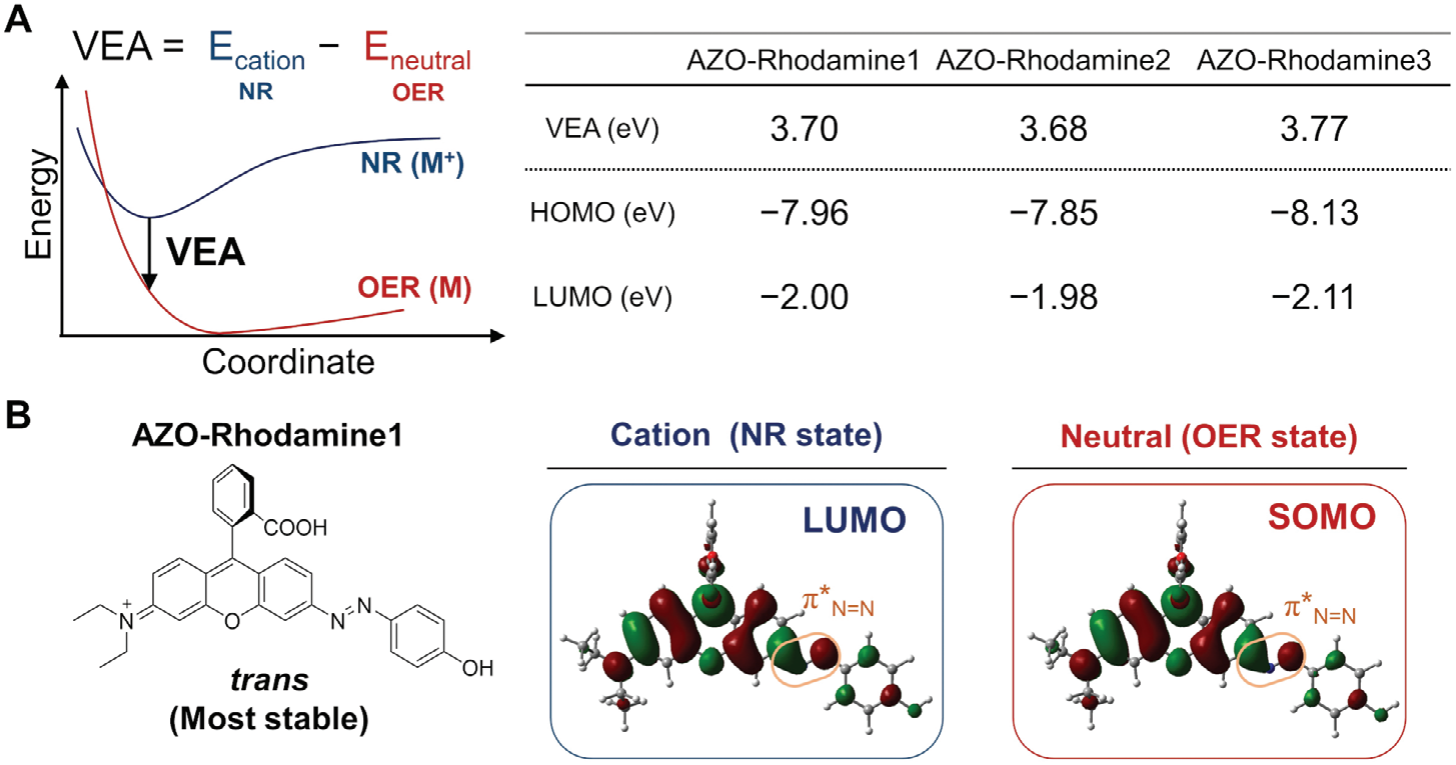
Quantum chemical calculations of AZO‐Rhodamine1‐3. Figure (A) displays the VEA and the LUMO energies of AZO‐Rhodamine1‐3. Figure (B) depicts the spatial distribution of the HOMO and LUMO of the NRstate and the SOMO of the OERstate of AZO‐Rhodamine1. The SOMO and LUMO are overlapping around the azo bond. All calculations were carried out using the Gaussian16 software at the ωB97XD/cc‐pVDZ/IEFPCM (Solvent = water) level.

The structures of the most stable conformers of AZO‐Rhodamine1‐3 obtained from the SC‐AFIR calculations by using the GRRM17 program are provided in the Supplementary Material. For all AZO‐Rhodamine1‐3, *trans* isomers were more stable than *cis* isomers. We evaluated the VEA [energy gap between non‐reduced (NR) and one‐electron‐reduced (OER) states at equilibrium structure of the NR form] of each compound by DFT calculations and compared it with the vertical binding energy (VBE) of hydrated electrons, which corresponds to the stabilization energy of the hydrated electrons. If the VEA of a compound is close to the VBE of hydrated electrons, it can be assumed that the reduction by hydrated electrons will be accelerated because of a resonance effect.^[^ ^19^ ^]^ Experimentally observed VBE values of hydrated electrons have been reported to be in the 3.4–3.7 eV range.^[^ ^20^, ^21^ ^]^ The VEAs of AZO‐Rhodamine1‐3 were calculated to be around 3.7 eV for all compounds (**Figure** **3**A), in close agreement with the previously reported VBE value of 3.7 eV. The LUMOenergies of AZO‐Rhodamine1−3 were ≈ −2.0 eV, suggesting that the reduction potential is almost the same among these compounds (Figure 3A). Subsequently, for each AZO‐Rhodamine compound, the spatial distribution of the LUMO in the NR state of the most stable conformer was compared to that of the single occupied molecular orbital (SOMO) in the OER state to determine whether the hydrated electrons could bind to the antibonding orbital of the azo bond, leading to azo bond cleavage. The LUMO of AZO‐Rhodamine1 was found to be a π^*^ orbital located around the Rhodamine moiety and azo bond. Similarly, the SOMO of the OER state was also located around the Rhodamine moiety and azo bond (Figure 3B). These results suggest that the reducing electron is first attached around the Rhodamine moiety or azo bond, and then it is transferred to the antibonding π^*^ orbital of the azo bond, resulting in azo bond cleavage.

### 2.4 X‐Ray Induced Azo Bond Cleavage in Living Cells

**Figure 4:**
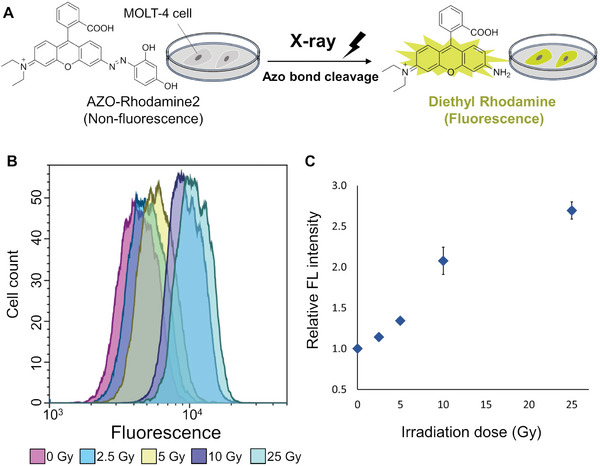
A) Method for measuring azo bond cleavage in cells. By incorporating azo bonds into fluorescent materials, their fluorescence could be lost. It has been shown that azo bonds can be cleaved by X‐ray irradiation, thus the cleavage of azo bonds inside cells can be evaluated by measuring the fluorescence of Diethyl Rhodamine produced upon X‐ray irradiation. (B) and (C) show the quantitative analysis of released Diethyl Rhodamine in cells measured by flow cytometry. B) Histogram of flow cytometer. Cells were irradiated at 0, 2.5, 5, 10, and 25 Gy. C) Relative fluorescence intensity obtained from G‐means of (B). Error bars represent the SEM (n = 3).

It is well known that when an azo bond is incorporated into a π‐conjugated system of a fluorophore, it loses its fluorescent properties due to the fact that the vibrational and rotational relaxation processes of the azo bond,^[^ ^22^ ^]^ which occur in the picosecond order_,_^[^ ^23^ ^]^ are faster than fluorescence of the nanosecond order. Therefore, azo bond cleavage can be detected in cells by comparing the fluorescence emitted from cells treated with AZO‐Rhodamine1‐3 before and after X‐ray irradiation (**Figure** **4**A). Since more Diethyl Rhodamine was released from AZO‐Rhodamine2, we selected this compound as a marker for azo bond cleavage inside the cells.

MOLT‐4 cells, frequently used to evaluate the effect of X‐ray irradiation,^[^ ^24^ ^]^ were incubated in an RPMI‐1640 medium containing 5 µM of AZO‐Rhodamine2 for 80 min. To confirm that the azo bond cleavage is only an intracellular event, the medium was replaced with phosphate buffer saline and the cells were irradiated with 2.5–25 Gy X‐rays after 10 min of nitrogen flushing at 4 °C. The fluorescence intensity from the cells was measured by flow cytometry. An increase in fluorescence intensity was observed at the lowest dose of 2.5 Gy, which further increased in a dose‐dependent manner up to an irradiation dose of 25 Gy (Figure 4B,C). These results suggested that the azo bond was cleaved by X‐ray irradiation under intracellular conditions.

## 3 Discussion

In this study, we investigated whether rationally designed azo compounds could be cleaved upon X‐ray irradiation and elucidated the corresponding reaction mechanism. AZO‐Rhodamine1‐3, which possesses a cationic charge and long π‐conjugate system, was synthesized. AZO‐Rhodamine2 was obtained by introducing an additional hydroxyl group at the meta‐position to the azo bond of AZO‐Rhodamine1. Furthermore, AZO‐Rhodamine3 was also generated by substituting a carboxylic group at the meta‐position to the azo bond of AZO‐Rhodamine1. The cationic charge shall enhance the electrostatic interactions with hydrated electrons that have a negative charge. In addition, a long π‐conjugated system makes the azo compound more reducible because hydrated electrons have a low ORP. Moreover, since the long π‐conjugated system of the designed compounds is directly linked to the azo bond, it can be expected that the azo bond will be selectively cleavable.

The azo bond was selectively cleaved by X‐ray irradiation as shown in Figure 1. In addition, a two‐electron reduced compound was detected in the AZO‐Rhodamine1 solution after X‐ray irradiation, which suggested that the azo bond cleavage reactions proceeded via a two‐electron reduction process. The results of quantum chemical calculations showed that the LUMO at the ground state and the SOMO at the one‐electron reduced state of AZO‐Rhodamine1 were both located around the azo bond to the Diethyl Rhodamine moiety. This supports that the azo bond could be reduced by a hydrated electron, resulting in azo bond cleavage. The efficiency of the reduction process of the azo bond was predicted to be the same among AZO‐Rhodamine1‐3 by quantum calculations since both VEA and LUMO did not change among AZO‐Rhodamine1‐3.

**Scheme 2:**
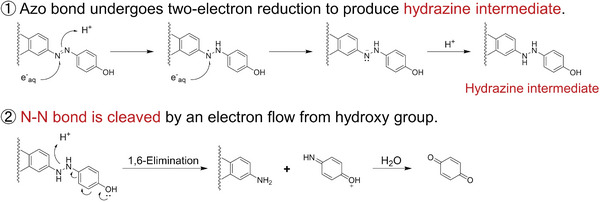
Proposed mechanism of azo bond cleavage by X‐ray irradiation.

In contrast to the reduction process, the reaction efficiency after the two‐electron reduction process was different. While no significant difference was observed with respect to the amount of reacted AZO‐Rhodamine1‐3, the amount of released Diethyl Rhodamine varied noticeably. Based on these results, the reaction mechanism of azo bond cleavage by X‐rays was assumed to consist of the following two‐step reaction (**Scheme** **2**). In the first step, the azo bond undergoes a two‐electron reduction with hydrated electrons generated by X‐ray irradiation, resulting in the formation of a hydrazine intermediate. In the second step, the N─N single bond of the hydrazine intermediate is cleaved by an electron flow from the electron‐donating functional group with a lone pair at the benzene moiety. Herein, a quinone imide produced by this reaction would react with water to form a quinone.^[^ ^25^ ^]^ According to a previous report, hydrazine N─N bonds were only cleaved when the benzene moiety of the hydrazine carried an electro‐donating functional group with a lone pair,^[^ ^22^ ^]^ which is consistent with our proposed mechanism. It should be noted that AZO‐Rhodamine2 possesses a hydroxyl group not only at the para‐position but also at the ortho‐position, resulting in efficient electron flow, which causes 1,4 or 1,6‐elimination and consequently releasing the greatest amount of Diethyl Rhodamine.

Considering the concentration of the generated hydrated electrons (5.4 µM at 20 Gy) and the two‐electron reduction mechanism, a maximum amount of released Diethyl Rhodamine by X‐ray was estimated to be 2.7 µM. By setting 2.7 µM to 100% yield, it was found that the produced Diethyl Rhodamine from AZO‐Rhodamine2 was more than 100% (105 ± 4.8%). Thus, the reaction efficiency of AZO‐Rhodamine2 was not inferior to previously reported X‐ray activatable caged compounds.^[^ ^15^, ^16^ ^]^ In contrast to AZO‐Rhodamine2, the yields of AZO‐Rhodamine1 and 3 were 24 ± 0.7% and 17 ± 0.3%, respectively. It was suggested that the difference in the functional group at the benzene moiety affected the Diethyl Rhodamine releasing efficiency. Therefore, it would be effective to design compounds incorporating other electron‐donating functional group in order to cleave azo bonds more efficiently.

We conducted further experiments in cultured cells to investigate whether the azo bond could be also cleaved intracellularly using AZO‐Rhodamine2. As a result, the fluorescence emitted by cells after X‐ray irradiation increased in a dose‐dependent manner, indicating the release of Diethyl Rhodamine. Hydrated electrons generated in cells are likely to be scavenged by amino acids or proteins; for example, human serum albumin helps keep cells from being damaged by allowing disulfide bonds to react with the hydrated electrons.^[^ ^26^ ^]^ Our results of cell studies suggested that the azo bond cleavage of AZO‐Rhodamine2 was likely faster than the scavenging speed of amino acids or proteins. Our data will help to design new caged compounds that are activated by X‐rays.

## 4 Conclusion

Our study revealed that azo compounds designed to enhance the reaction efficiency with hydrated electrons could be efficiently cleaved by X‐ray irradiation. It was hypothesized that the azo bond cleavage mechanism takes place in two steps; 1) two‐electron reduction of the azo bond and 2) subsequent N─N bond cleavage caused by an electron flow from a lone pair. This hypothesis was supported by X‐ray irradiation and quantum chemical calculation results. Furthermore, cellular experiments also suggested that the azo bond cleavage occurred within the cell. Since this study clearly shows a proof‐of‐concept, we plan in the future to develop molecules that release anti‐cancer drugs triggered by azo bond cleavage upon X‐ray irradiation and investigate differences in the effect between normal and cancer cells.

## Conflict of Interest

The authors declare no conflict of interest.

## Supporting information

Supporting Information

## Data Availability

The data that support the findings of this study are available from the corresponding author upon reasonable request.
