# Supplementary material for: Theoretical Design and Synthesis of Caged Compounds Using X‐Ray‐Triggered Azo Bond Cleavage
Source: Adv Sci (Weinh). 2024 Jan 15;11(12):2306586. doi: 10.1002/advs.202306586 (PMC10966529; doi:10.1002/advs.202306586)
Supplement: Supplementary file 1 — Supporting Information [file ADVS-11-2306586-s001.pdf]

ADVANCED SCIENCE
Open Access

Supporting Information

for *Adv. Sci.*, DOI 10.1002/advs.202306586

Theoretical Design and Synthesis of Caged Compounds Using X-Ray-Triggered Azo Bond Cleavage

*Koki Ogawara, Osamu Inanami, Hideo Takakura, Kenichiro Saita, Kohei Nakajima, Sonu Kumar, Naoya Ieda, Masato Kobayashi, Tetsuya Taketsugu and Mikako Ogawa**

Supporting Information
©Wiley-VCH 2021
69451 Weinheim, Germany

Theoretical design and synthesis of caged compounds using X-ray-triggered azo bond cleavage

Koki Ogawara, Osamu Inanami, Hideo Takakura, Kenichiro Saita, Kohei Nakajima, Sonu Kumar, Naoya Ieda, Masato Kobayashi, Tetsuya Taketsugu, Mikako Ogawa

Abstract: Caged compounds are frequently used in life science research. However, the light used to activate them is commonly absorbed and scattered by biological materials, limiting their use to basic research in cells or small animals. In contrast, hard X-rays exhibit high bio-permeability due to the difficulty of interacting with biological molecules. With the main goal of developing X-ray activatable caged compounds, we designed and synthesized azo compounds with a positive charge and long π-conjugated system to increase the reaction efficiency with hydrated electrons. The azo bonds in the designed compounds were selectively cleaved by X-ray, and the fluorescent substance Diethyl Rhodamine was released. Based on the results of experiments and quantum chemical calculations, azo bond cleavage was assumed to occur via a two-step process: a two-electron reduction of the azo bond followed by N-N bond cleavage. Cellular experiments also demonstrated that the azo bonds could be cleaved intracellularly. Thus, we successfully generated caged compounds that can be activated by an azo bond cleavage reaction promoted by X-ray.

Table of Contents

Experimental Procedures 3
Reagents and general information 3
Azo bond cleavage by X-ray irradiation 3
Quantum chemical calculations 3
Dose dependency for Diethyl Rhodamine released from AZO-Rhodamine2 4
Cell study 4
Synthetic procedure 5
Diethyl Rhodamine 5
AZO-Rhodamine1 5
AZO-Rhodamine2 6
AZO-Rhodamine3 6
Figure and table 7
Figure S1. H^1-NMR spectrum of AZO-Rhodamine1. 7
Figure S2. ^{13}C-NMR spectrum of AZO-Rhodamine1. 8
Figure S3. High-resolution spectrum of AZO-Rhodamine1. 9
Figure S4. H^1-NMR spectrum of AZO-Rhodamine2. 10
Figure S5. ^{13}C-NMR spectrum of AZO-Rhodamine2. 11
Figure S6. High-resolution spectrum of AZO-Rhodamine2 12
Figure S7. H^1-NMR spectrum of AZO-Rhodamine3. 13
Figure S9. High-resolution spectrum of AZO-Rhodamine3. 15
Figure S10. Raw data of the LC-MS chart of AZO-Rhodamine1 after irradiation. 16
Figure S11. Raw data of the LC-MS chart of AZO-Rhodamine2 after irradiation. 17
Figure S12. Raw data of the LC-MS chart of AZO-Rhodamine3 after irradiation. 18
Table S1. Cartesian coordinates (in Å) of the lowest energy (most stable) structure of AZO-Rhodamine1 in the NR state.. 19
Table S2. Cartesian coordinates (in Å) of the lowest energy (most stable) structure of AZO-Rhodamine2 in the NR state.. 20
Table S3. Cartesian coordinates (in Å) of the lowest energy (most stable) structure of AZO-Rhodamine3 in the NR state. 21
References 23
Author Contributions 24

Experimental Procedures

Reagents and general information

AZO-Rhodamine1–3 were synthesized by azo coupling of Diethyl Rhodamine. Please see the protocol in the synthetic procedure for details. High performance liquid chromatography (HPLC) analyses were carried out on an HPLC system (Shimadzu Corporation, Kyoto, Japan) equipped with a reverse-phase column Inertsil ODS-3 (4.6 mm × 250 mm) (GL Sciences Inc. Tokyo, Japan), using eluent A (0.1% trifluoroacetic acid (TFA) aqueous solution) and eluent B (99% CH_3CN containing 1% H_2O) with a flow of 1 mL/min. Liquid chromatography-mass spectrometry (LC-MS) analyses were carried out on an LC-MS system (Waters Xevo G2-XS Q-tof) (Waters Corporation, MA, USA) equipped with a reverse-phase column, i.e., ACQUITY UPLC® BEH C18 column (2.1 mm × 50 mm) (Waters Corporation), using eluent A (1% sodium formate buffer aqueous solution) and eluent B (99% CH_3CN containing 1% H_2O). X-rays were generated using either a CLINAC (Varian Medical Systems, Palo Alto, CA, USA) or an X-Rad iR-225 (Precision X-Ray, North Branford, CT, USA) machine. Emission spectra of compounds were measured using a spectrophotometer FP-8550 (JASCO, Tokyo, Japan). ^{1}H-NMR spectra were recorded on either a JNM-ECX400P or JNM-ECS400 (JEOL, Tokyo, Japan) at 400 MHz. Chemical shifts were referred to tetramethyl silane or heavy solvent peak as standard and expressed in ppm values.

In the cell study, intracellular fluorescence was measured using a CytoFlex (Beckman Coulter, Pasadena, CA, USA) with a set excitation wavelength of 488 nm and fluorescence wavelength of 564‑606 nm.

Azo bond cleavage by X-ray irradiation

Each 5 μM solution of AZO-Rhodamine1–3 in 1 mM phosphate buffer (pH 7.4) containing 40% MeOH (due to the low solubility of AZO-Rhodamine1–3) was prepared in a vial. Since MeOH has been reported to act as a hydroxyl radical scavenger, we can mainly evaluate the reaction caused by hydrated electrons under these conditions.[1] The solution was bubbled with argon gas through the septum cap of a sealed vial to remove oxygen and irradiated with X-ray at 20 Gy using CLINAC (irradiation dose rate: 4.7 Gy/min, tube voltage: 6 MeV). Each irradiated solution was mixed with phosphate buffer (pH 7.4, 1% DMF) containing 1 μM Rhodamine B as an internal standard with the same volume and analyzed by HPLC (B conc: 40% (0 min) → 40% (4 min) → 100% (13 min), Injection volume = 150 μL, wavelength = 538 or 558 nm) and LC-MS (B conc: 20% (0min) → 20% (2 min) → 100% (5.5 min), Injection volume = 5 μL) .

Quantum chemical calculations

Density functional theory (DFT) calculations were carried out with a Gaussian16 program,[2] using the long-range corrected ωB97XD functional (including Grimme's dispersion correction) and cc-pVDZ basis set. The solvent effect of water was taken into account by using the integral equation formalism variant of the polarizable continuum model (IEFPCM). For each AZO-Rhodamine derivative, equilibrium structures were systematically searched by the single-component artificial force induced reaction (SC-AFIR) method implemented in GRRM17 software,[3] and then the lowest energy structure was employed for the vertical electron affinity (VEA) calculation. The VEA is calculated as the energy gap between non-reduced (NR) and one-electron-reduced (OER) states at equilibrium

structure of the NR form (see Figure 3A in the main text).

Dose dependency for Diethyl Rhodamine released from AZO-Rhodamine2

A 5 μM AZO-Rhodamine2 solution in 1 mM phosphate buffer (pH 7.4) containing 40% MeOH was prepared in a vial. The solution was bubbled with argon gas through the septum cap of a sealed vial and irradiated with X-rays at 4, 10, 20, and 40 Gy using CLINAC (irradiation dose rate: 4.7 Gy/min, tube voltage: 6 MeV). Subsequently, fluorescence spectra of these solutions were measured (excitation wavelength: 480 nm).

Cell study

Human leukemia cell lines MOLT-4 were purchased from RIKEN Cell Bank (Tsukuba, Japan). MOLT-4 cells were cultured in an RPMI-1640 medium supplemented with 10% fetal bovine serum and 1% penicillin/streptomycin and maintained in a humidified atmosphere containing 5% CO_2 at 37 °C. The cells were sub-cultured when the cell concentration reached 1.0×10^4 cells/mL.

To prepare the cell suspensions for X-ray irradiation experiments, 3.0×10^7 MOLT-4 cells were suspended in 30 mL of an RPMI-1640 medium containing 10% fetal bovine serum (1.0×10^6 cells/mL). Then, 150 μL of 1 mM AZO-Rhodamine2 methanol solution was mixed with 600 μL of RPMI-1640 medium containing the MOLT-4 cells. Next, this 750 μL of RPMI-1640 medium containing AZO-Rhodamine2 was dissolved 29250 μL of RPMI-1640 medium. This stepwise dilution ensured that AZO-Rhodamine2 did not form a precipitate in the medium.

After incubating the cells with the dye solution for 80 min, the medium was removed by centrifugation at 400 g for 5 min, and the cells were resuspended in 30 mL of phosphate-buffered saline (final cell concentration: 1.0×10^6 cells/mL). The cell suspensions were transferred to sealed vials and bubbled with nitrogen gas for 10 min to remove oxygen, and further irradiated with X-rays at various doses (0, 2.5, 5, 10, and 25 Gy) setting the dose rate at 4.37 Gy/min. Low-energy X-rays were filtered out using a 0.3 mm copper plate. Each irradiated sample was transferred to a measuring tube and analyzed using CytoFlex.

Synthetic procedure

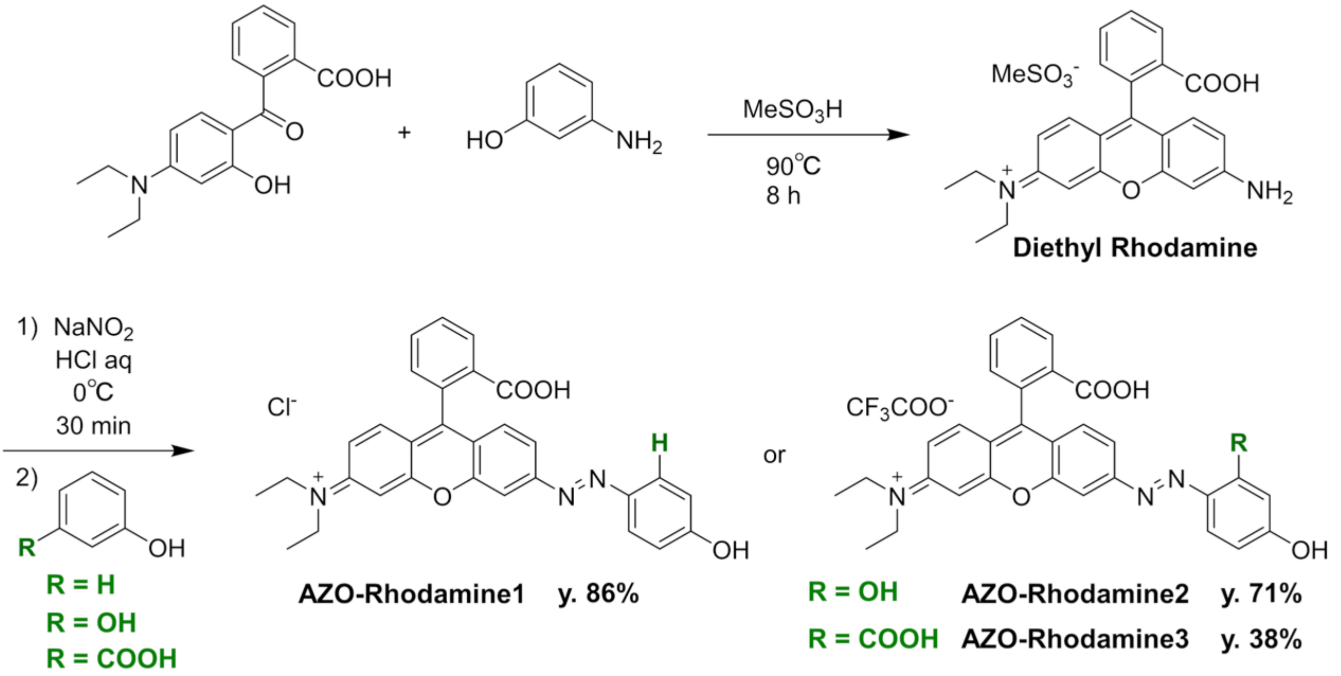

Diethyl Rhodamine

2-(4-Dietylamino-2-hydroxylbenzoyl) benzoic acid (2.19 g, 7.00 mmol), 3-aminophenol (763 mg, 7.00 mmol) were added to methane sulfonic acid (10 mL) in a round bottle flask and reacted at 90 °C for 7 h. The reaction solution was then dissolved in RO water and salted out using potassium chloride. The salted product was recrystallized using 100 mL of toluene to obtain red crystals of the target compound (982 mg). The yield was 29%. No further purification was performed.

AZO-Rhodamine1

Diethyl Rhodamine methane sulfonic acid (100 mg, 0.207 mmol) was dissolved in RO water (8 mL), acetonitrile (8 mL), and 1 M hydrochloric acid (4 mL) in the round bottle flask. After cooling, sodium nitrite (28.6 mg, 0.414 mmol) was added and the reaction mixture was stirred at 0 °C for 15 min. Then, phenol (39 mg, 0.415 mmol) was dissolved in MeOH (2 mL), and this MeOH solution was added dropwise to the previous mixture and allowed to react over 15 min. Next, 0.1 M aq. ammonia was added until pH 6–7 was reached. After removal of all the volatiles under reduced pressure, the residue was purified by column chromatography using silica gel (methanol: dichloromethane = 1:50) to afford the target compound (90.3 mg). The yield was 86%; (^{1}H-NMR Spectra 400 MHz, $CDCl_3$): δ 8.06 (d, *J* = 7.6 Hz, 1H), 7.80 (d, *J* = 12.0 Hz, 1H), 7.73-7.61 (m, 3H), 7.47 (d, *J* = 8.8 Hz, 1H), 7.22 (d, *J* = 8.0 Hz, 1H), 6.92 (d, *J* = 9.2 Hz, 2H), 6.86 (d, *J* = 8.4 Hz, 1H), 6.62 (d, *J* = 9.2 Hz,1H), 6.50 (s, 1H), 6.39 (d, *J* = 9.2, 1H), 3.70 (q, *J* = 7.2 Hz, 4H), 1.18 (t, *J* = 7.2 Hz, 6H); (^{13}C-NMR Spectra 101 MHz, $CDCl_3$): δ 170.4, 159.6, 154.4, 153.6, 153.5, 152.8, 150.3, 147.4, 135.5, 130.1, 129.3, 129.1, 125.8, 125.5, 124.6, 121.2, 118.1, 116.4, 111.3, 109.8, 105.1, 98.1, 84.8, 45.0, 13.0; HRMS (ESI) $C_{30}H_{25}N_3O_4$: $[M]^+$ calcd. for 492.1918, found, 492.1916 (–0.2 mmu); Anal. Calcd. for $C_{30}H_{28}N_3O_5Cl{\bullet}H_2O$: C; 65.99%, H; 5.17%, N; 7.70%, found C; 66.08%, H; 4.91%, N; 7.45%.

AZO-Rhodamine2

Diethyl Rhodamine methane sulfonic acid (100 mg, 0.207 mmol) was dissolved in RO water (16 mL), acetonitrile (16 mL), and 1 M hydrochloric acid (3 mL) in a round bottle flask, and the solution was cooled to 0 °C. Subsequently, sodium nitrite (28.6 mg, 0.414 mmol) was added and the mixture was allowed to react at 0 °C for 15 min. Then, resorcinol (45.6 mg, 0.415 mmol) was dissolved in MeOH (2 mL), and this methanol solution was added dropwise to previous solution and the mixture was allowed to react for 15 min. Next, 0.1 M aq. ammonia was added until pH 6–7. After removal of all the volatiles under reduced pressure, the residue was purified by column chromatography using silica gel (dichloromethane/ methanol = 1:50). Further purifications were carried out by reverse phase HPLC using aq. TFA 0.1% and acetonitrile as mobile phase (Acetonitrile conc: 30% (0 min) → 30% (2 min) → 50% (4 min) → 55% (13 min) → 60% (15 min)), evaporated and target compound were obtained (72.7 mg). The yield was 71 %; (^{1}H-NMR Spectra 400 MHz, CD_3OD): δ 8.40 (d, *J* = 7.6 Hz, 1H), 7.96-7.85 (m, *J* = 12.0 Hz, 3H), 7.74 (d, *J* = 9.2, 1H), 7.60 (d, *J* = 9.2 Hz, 1H), 7.51 (d, *J* = 9.2 Hz, 1H), 7.20 (dd, *J* = 9.2 Hz, 9.2 Hz, 2H), 7.03 (s, 1H), 6.55 (d, *J* = 9.2 Hz, 1H), 6.23 (s, 1H), 3.76 (q, *J* = 7.2 Hz), 1.37 (t, *J* = 7.2 Hz, 6H); (^{13}C-NMR Spectra 101 MHz, $CDCl_3$): δ 168.2, 167.7, 162.0, 160.3, 156.0, 155.8, 136.0, 135.2, 134.4, 133.5, 132.3, 131.9, 131.8, 131.3, 131.0, 122.8, 119.7, 118.9, 113.1, 109.8, 98.0, 47.9, 13.0; HRMS (ESI) $C_{30}H_{25}N_3O_5$: $[M]^+$ calcd for 508.1867, found, 508.1868 (+0.1 mmu); Anal. Calcd. for $C_{32}H_{26}N_3O_7F_3$ • 1/2CF_3COOH: C, 58.41%; H, 3.94%; N, 6.19%, found C, 58.79%; H, 3.98%; N, 6.25%.

AZO-Rhodamine3

Diethyl Rhodamine methane sulfonic acid (100 mg, 0.207 mmol) was dissolved in RO water (8 mL), acetonitrile (8 mL), and 1 M hydrochloric acid (3 mL) in the round bottle flask. After cooling, sodium nitrite (22 mg, 0.319 mmol) was added and the mixture was allowed to react at 0 °C for 15 min. Then, m-hydroxyl benzoic acid (36 mg, 0.295 mmol) was dissolved in acetonitrile (2 mL), and this acetonitrile solution was gradually added to the previous solution, and the stirring was continued for 15 min. Next, 0.1 M aq. ammonia was added until pH 6–7. After removal of all the volatiles under reduced pressure, the residue was purified by column chromatography on silica gel (dichloromethane: methanol = 90:10 → 50:50). Further purifications were carried out by reverse phase HPLC using TFA 0.1% aq and acetonitrile as mobile phase (Acetonitrile conc: 42% (0 min) → 42% (7 min) → 85% (18 min)), evaporated and target compound were obtained (50.5 mg). The yield was 38%; (^{1}H-NMR Spectra 400 MHz, CD_3OD) : δ 8.27 (d, *J* = 7.8 Hz, 1H), 7.94 (s, 1H), 7.83-7.71 (m, 4H), 7.39 (d, *J* = 7.8 Hz, 1H), 7.24 (d, *J* = 8.8, 1H), 7.17 (s, 1H), 7.08 (s, 1H), 7.03 (s, 1H), 6.91 (d, *J* = 9.2 Hz, 1H), 3.70 (q, *J* = 7.2 Hz, 4H), 1.26 (t, *J* = 7.2 Hz, 6H); (^{13}C-NMR Spectra 101 MHz, $CDCl_3$): δ 146.1, 144.5, 137.2, 136.1, 135.5, 133.6, 125.1, 118.7, 117.4, 116.1, 115.1, 114.6, 114.3, 113.8, 108.7, 106.4, 106.2, 105.4, 104.2, 103.8, 103.0, 99.3, 83.3, 47.9, 20.0; HRMS (ESI) $C_{31}H_{26}N_3O_6$: $[M]^+$ calcd. 536.1816, found, 536.1828 (+1.2 mmu); Anal. Calcd. for $C_{33}H_{26}N_3O_8F_3$ • 1/2H_2O: C, 60.18%; H, 4.13%; N, 6.38%, found, C, 60.29%; H, 4.03%; N, 6.05%.

Figure and table

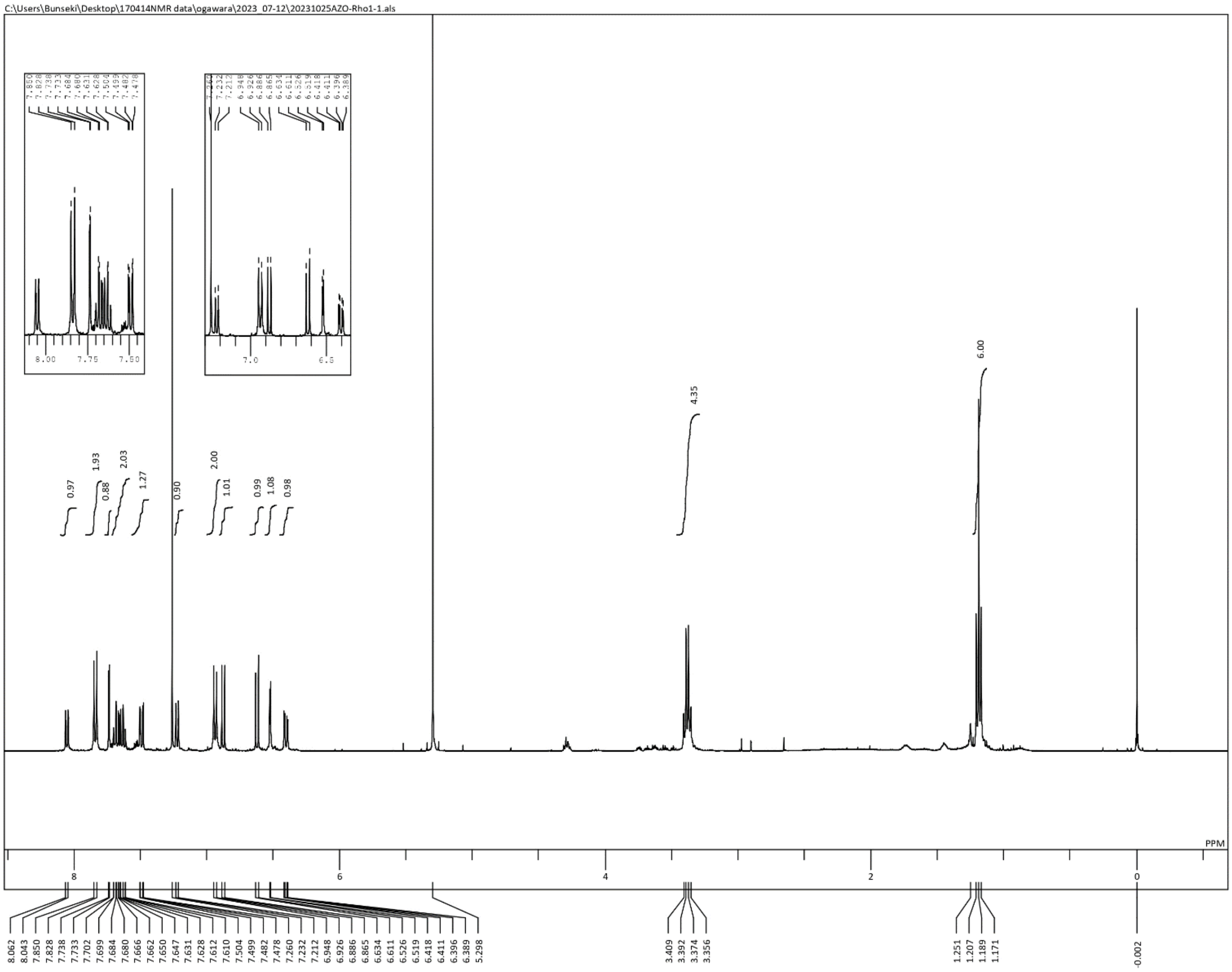

Figure S1. H[1]-NMR spectrum of AZO-Rhodamine1.

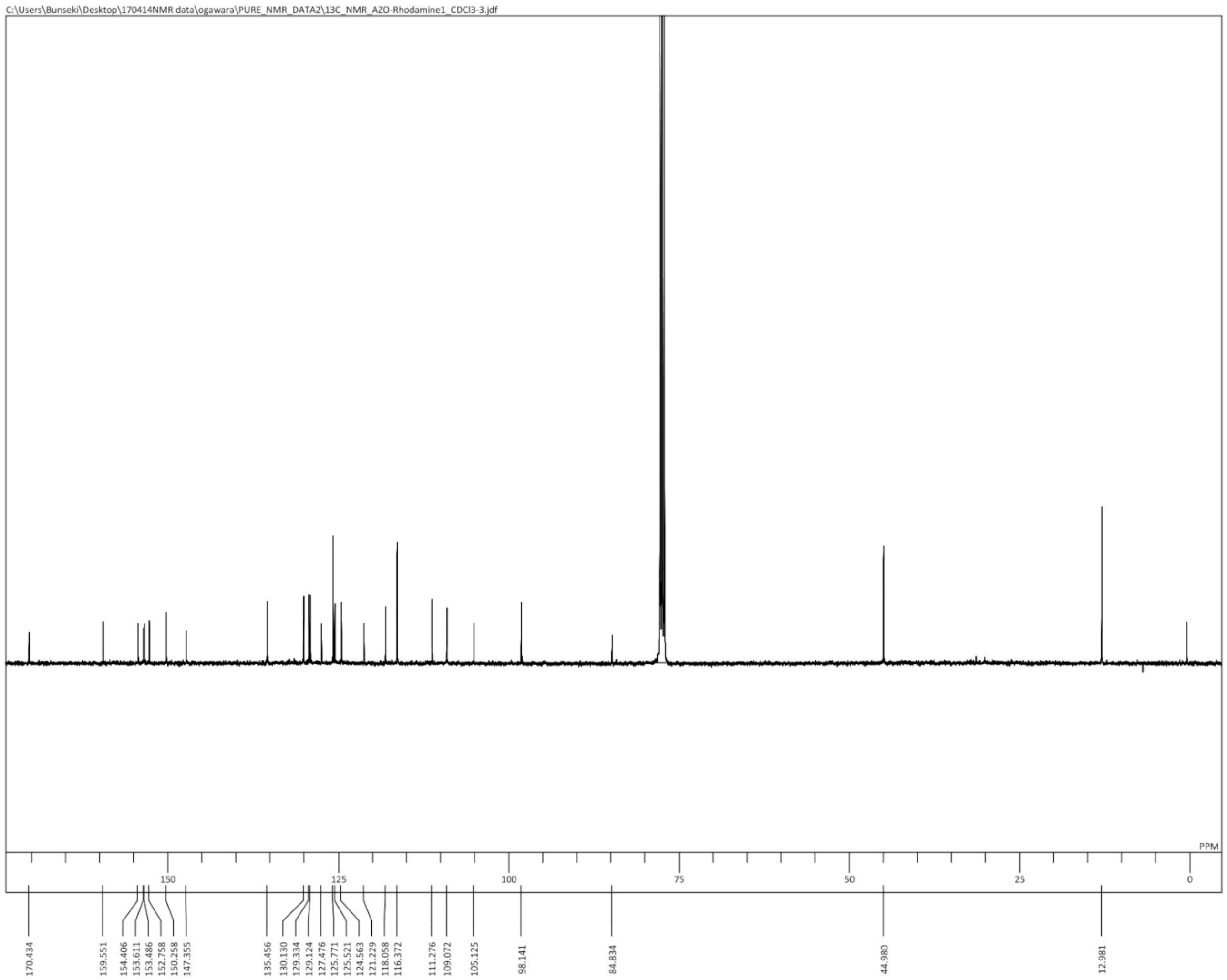

Figure S2. ^{13}C-NMR spectrum of AZO-Rhodamine1.

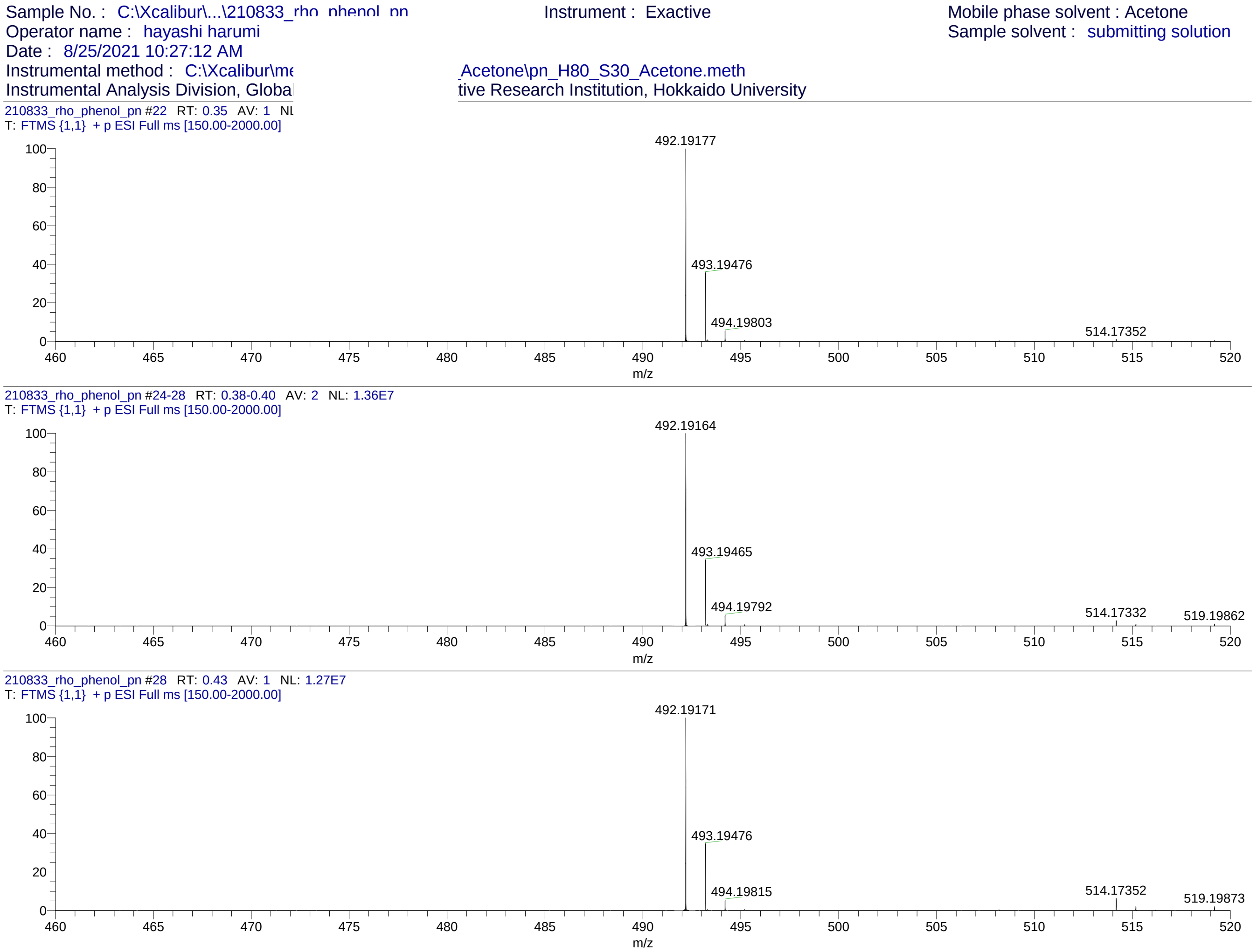

Figure S3. High-resolution spectrum of AZO-Rhodamine1.

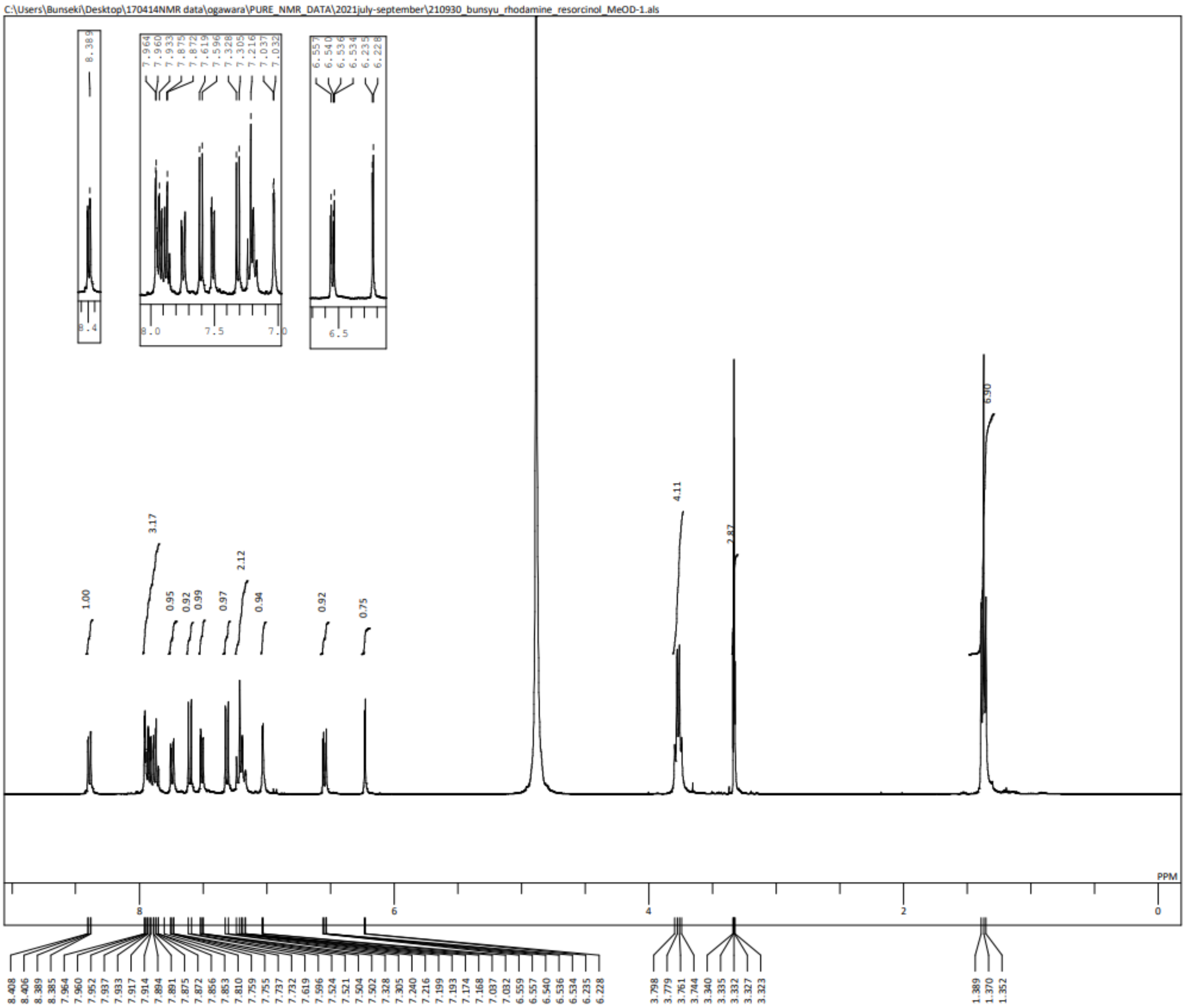

Figure S4. H1-NMR spectrum of AZO-Rhodamine2.

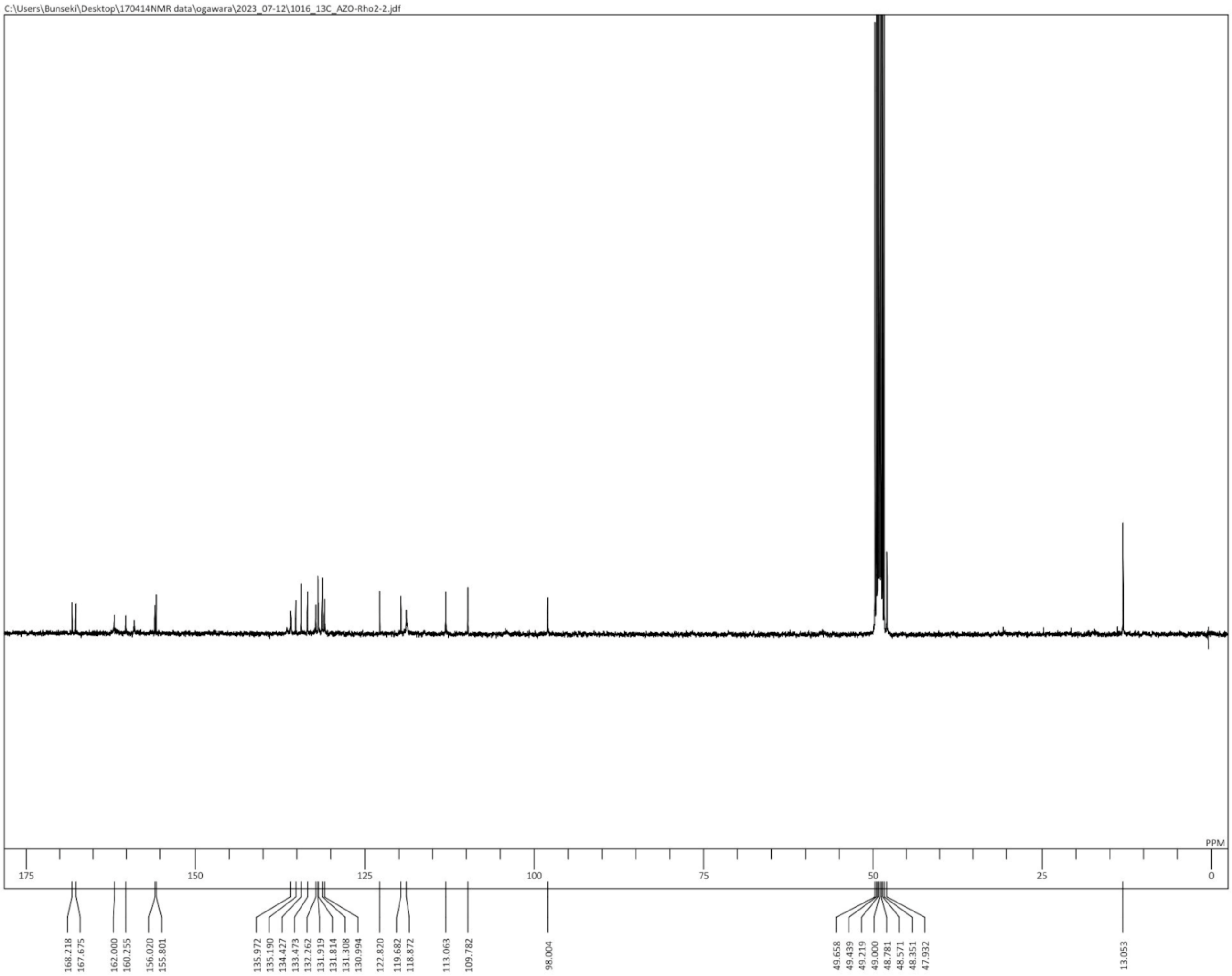

Figure S5. ^{13}C-NMR spectrum of AZO-Rhodamine2.

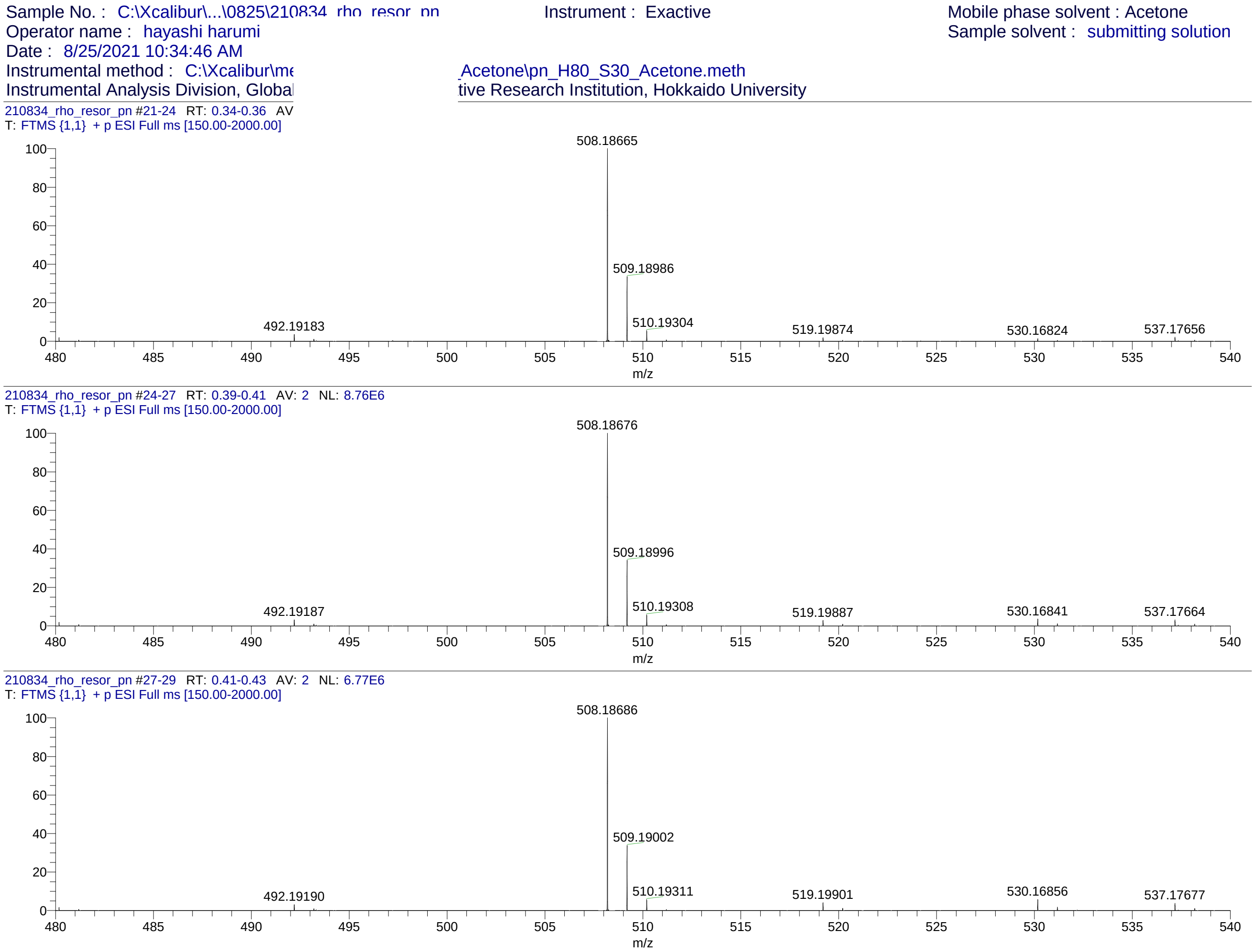

Figure S6. High-resolution spectrum of AZO-Rhodamine2

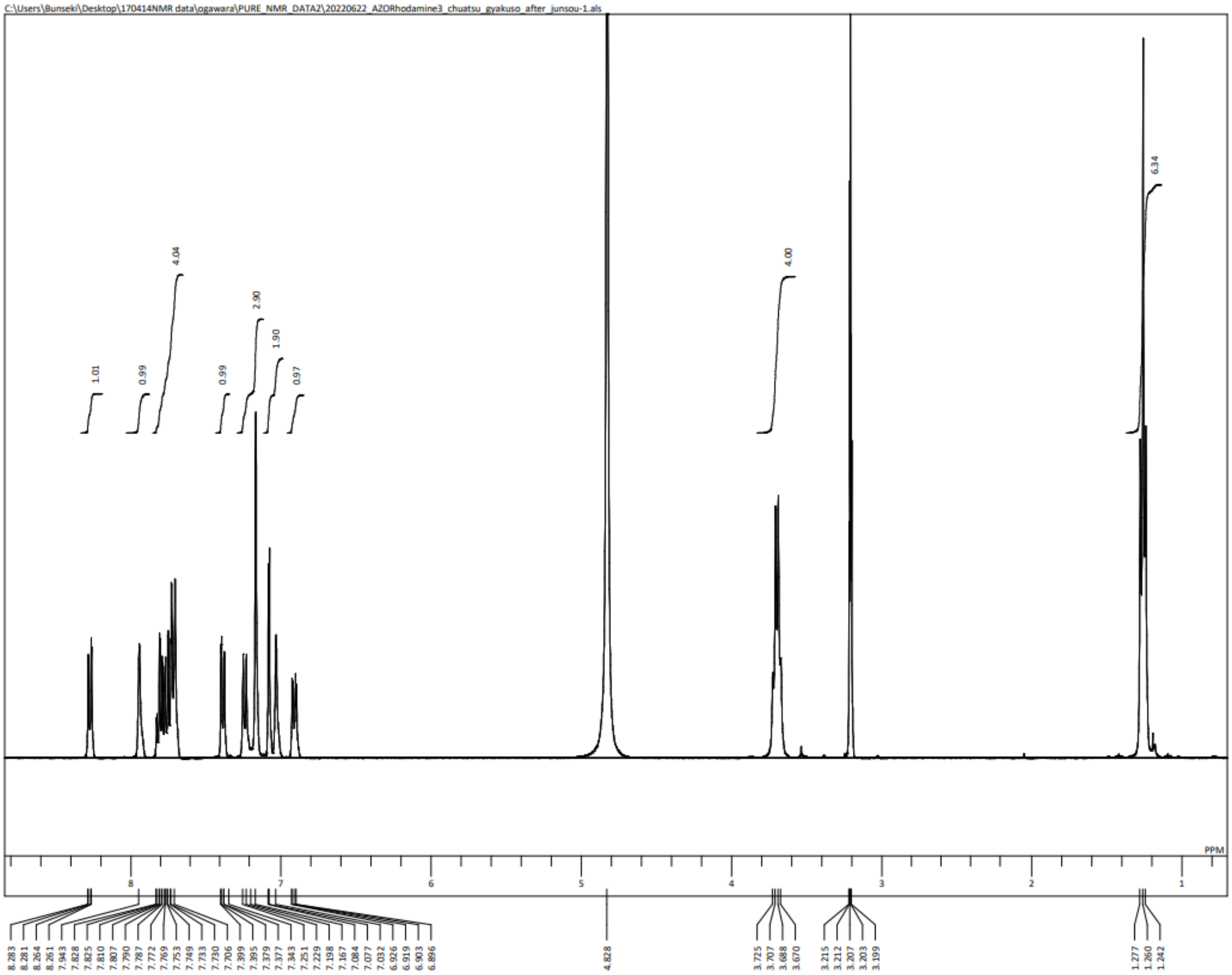

Figure S7. H^1-NMR spectrum of AZO-Rhodamine3.

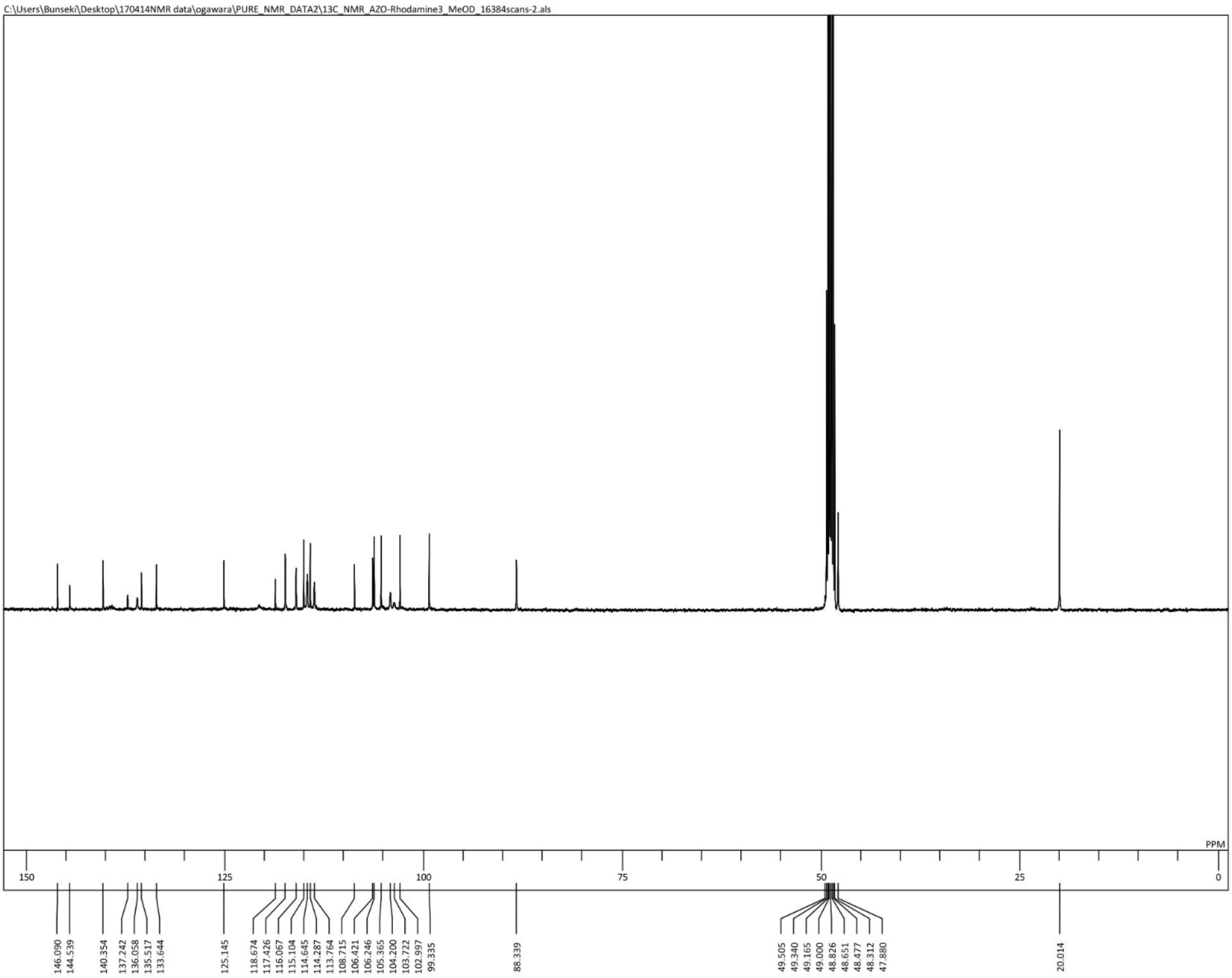

Figure S8. 13C-NMR spectrum of AZO-Rhodamine3.

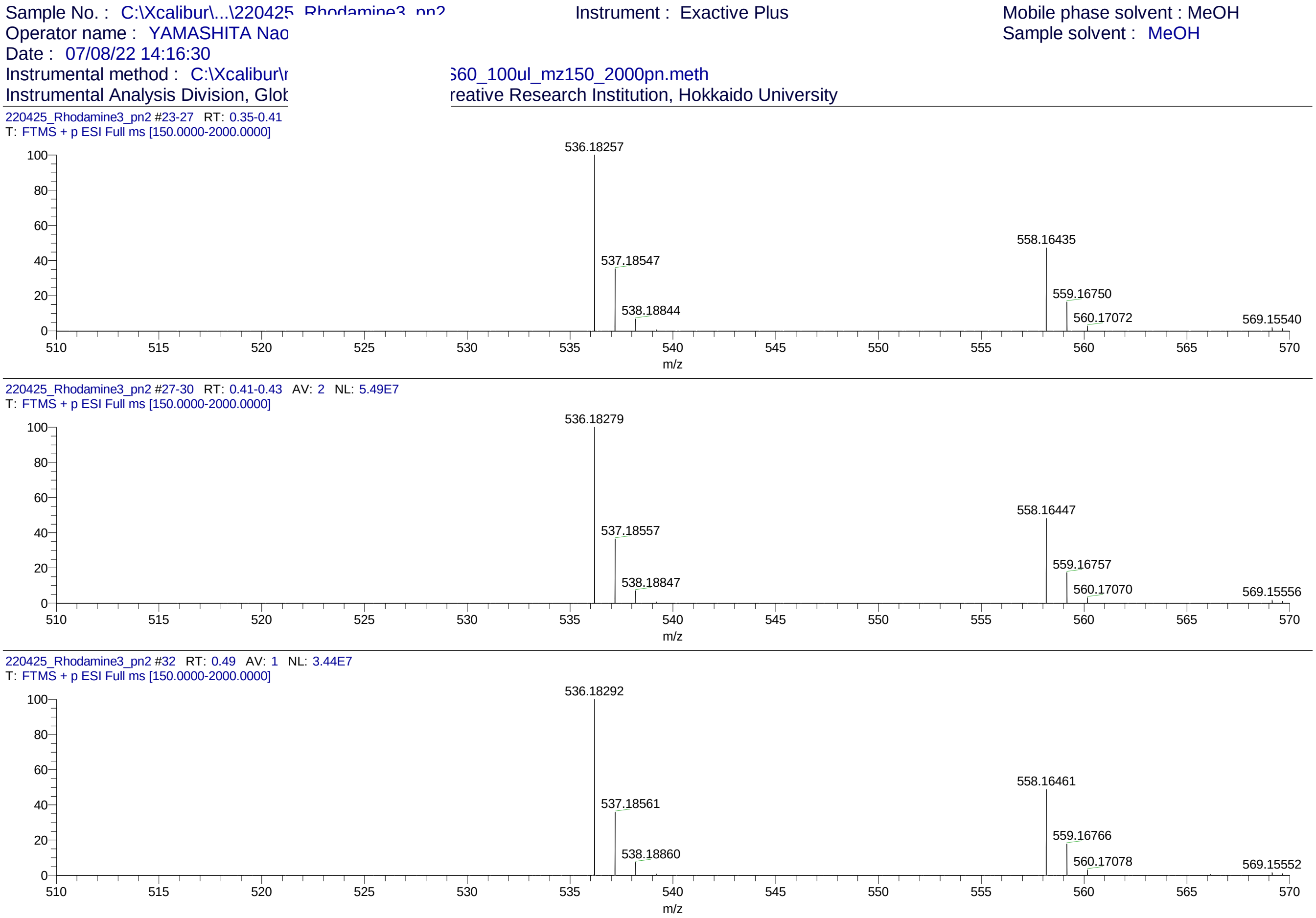

Figure S9. High-resolution spectrum of AZO-Rhodamine3.

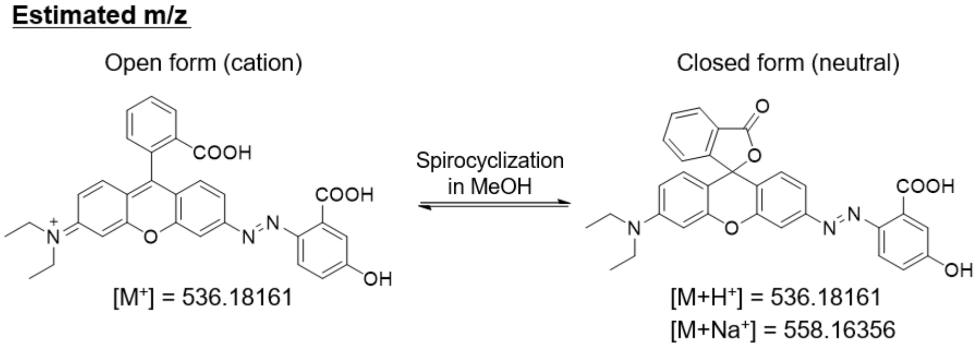

(The *m/z* = 558.164 is derived from spirocyclization products of AZO-Rhodamine3.)

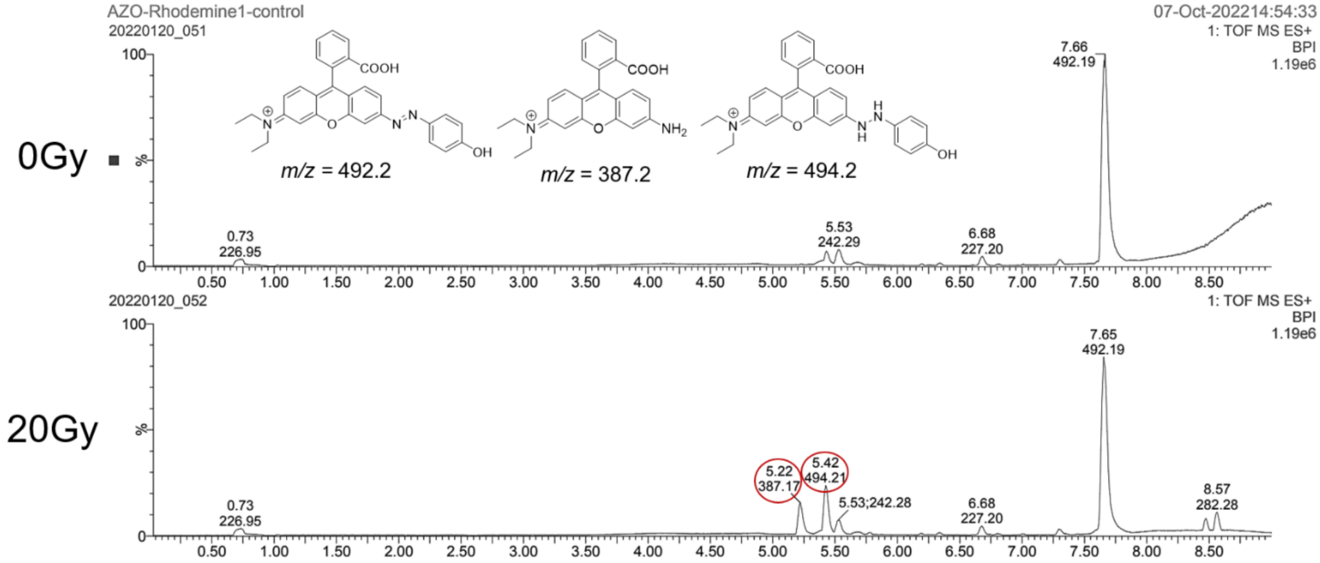

Figure S10. Raw data of the LC-MS chart of AZO-Rhodamine1 after irradiation.

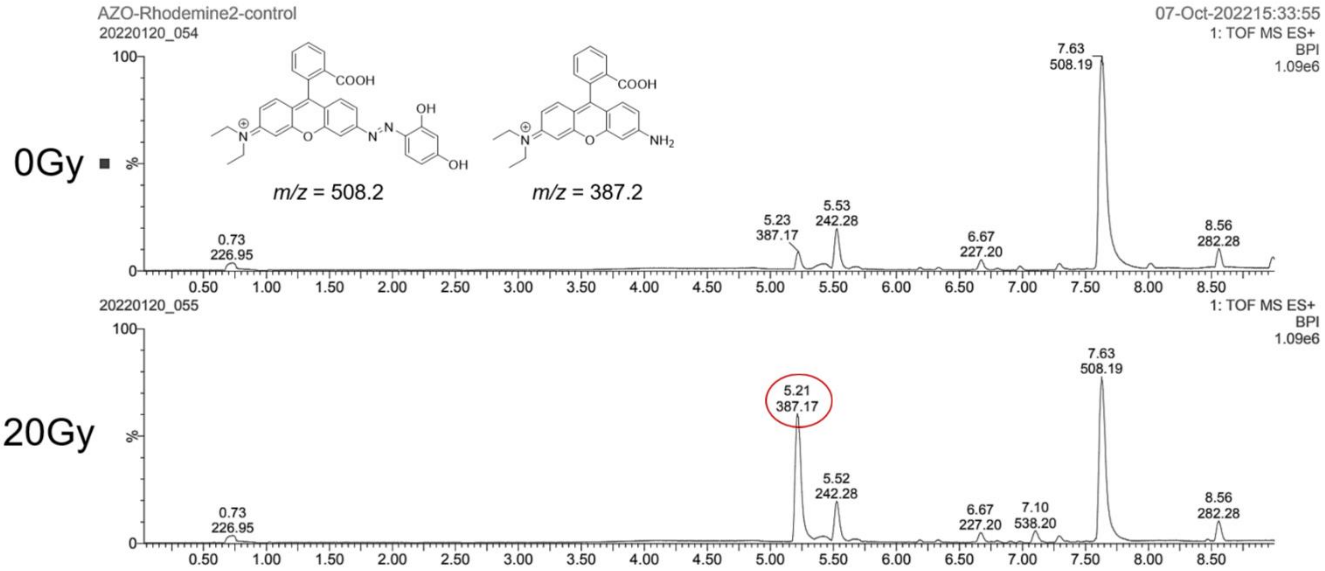

Figure S11. Raw data of the LC-MS chart of AZO-Rhodamine2 after irradiation.

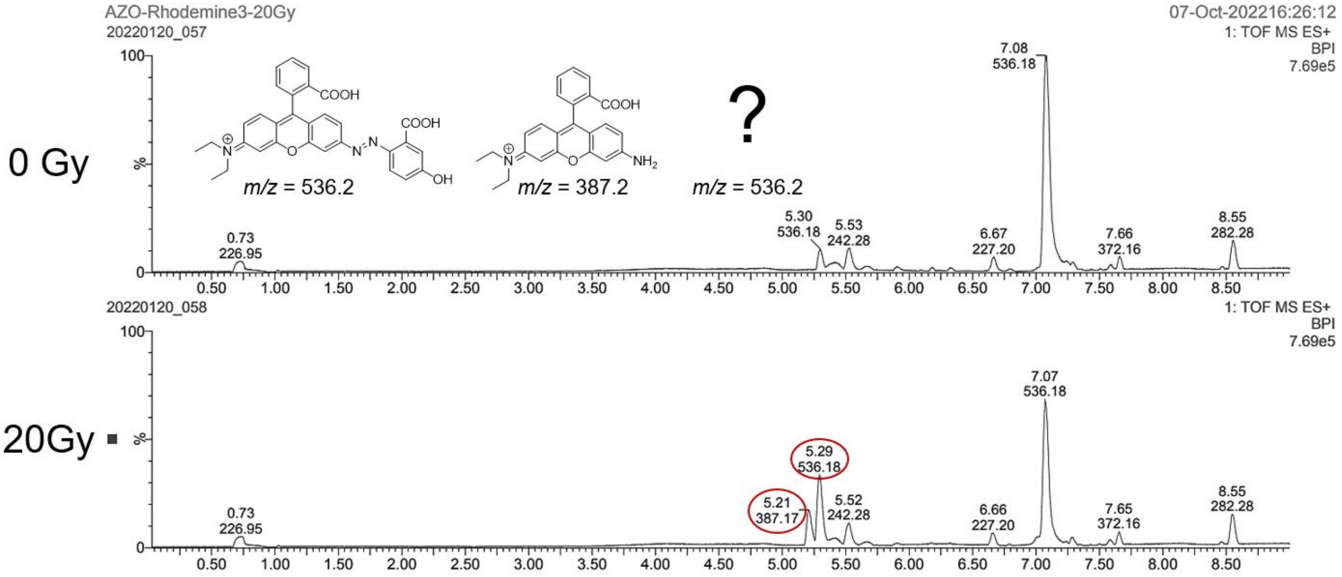

Figure S12. Raw data of the LC-MS chart of AZO-Rhodamine3 after irradiation.

Table S1. Cartesian coordinates (in Å) of the lowest energy (most stable) structure of AZO-Rhodamine1 in the NR state. The potential energy of the NR state (charge = +1, spin multiplicity = 1 [singlet]) is −44175.9193366963 hartree, and that of the OER state (charge = 0, spin multiplicity = 2 [doublet]) is −44179.6221391412 hartree. Computational level was ωB97XD/cc-pVDZ | IEFPCM(solvent = water).

Atom	x	y	z
O	-0.33056500	1.28443600	-0.06788900
O	-2.51066000	-4.04318900	2.69122300
O	-1.65312200	-2.04574700	2.15681100
N	-4.50872400	3.48131600	0.03240200
N	4.08483200	-0.63948200	-0.15197500
C	-1.53370600	-1.22463600	-0.37428200
C	-2.30598000	-0.08067600	-0.29694100
C	-0.10706200	-1.10178200	-0.32559700
C	-1.67105000	1.19238900	-0.12715700
C	0.45075900	0.18190900	-0.16854400
C	-3.79360200	2.35436700	-0.07614200
C	2.66636200	-0.71350800	-0.20433800
C	-2.15662200	-2.55574400	-0.62321000
C	-2.37497400	2.36757000	-0.02163000
C	1.82365800	0.38892400	-0.10725700
C	-3.73970700	-0.07476600	-0.34793500
C	0.77410000	-2.20094000	-0.42359500
C	-4.45125300	1.07457700	-0.24636400
C	2.13651800	-2.00970000	-0.36174800
C	-3.87402000	4.77727600	0.29011800
C	-5.96995600	3.50877900	-0.09714200
N	4.52718700	0.52680500	-0.05470700
C	-2.45567900	-3.46392900	0.40679000
C	-2.43688400	-2.89917300	-1.94744300
C	-3.43233400	5.47859800	-0.98906500
C	-6.68117300	3.25888100	1.22693400
C	-3.03177900	-4.69888000	0.09291600
C	-3.00851100	-4.13397000	-2.24887400
C	-3.30639400	-5.03501500	-1.22894500
C	-2.15998100	-3.09466900	1.82018700
H	-1.80649500	3.28703700	0.08567400
H	2.22662200	1.39251800	0.01469600
H	-4.26207100	-1.02425500	-0.46380400
H	0.36347000	-3.20271500	-0.54832300
H	-5.53622400	1.01949100	-0.27505500
H	2.82831000	-2.84860600	-0.43370200
H	-4.61033000	5.38976900	0.82778300
H	-3.03282800	4.63684400	0.98160600
H	-6.23082200	4.50058800	-0.49045300
H	-6.28443500	2.79022800	-0.86438600
H	-2.20358700	-2.19366400	-2.74611700
H	-2.68232100	4.88503200	-1.53209400
H	-4.28851600	5.64714100	-1.65881900
H	-2.98974900	6.45435100	-0.74356100
H	-6.45092500	2.25830300	1.62185300
H	-6.38089800	4.00241700	1.97998000
H	-7.76862500	3.33298900	1.08443100
H	-3.26404500	-5.39567600	0.89702500
H	-3.22160000	-4.38878900	-3.28774000
H	-3.75491900	-6.00110700	-1.46219400
H	-2.28179500	-3.72010000	3.57784100
C	5.92812600	0.63824000	0.00494300
C	6.81988300	-0.44907000	-0.03199900
C	6.43186100	1.93956200	0.10828500
C	8.18257300	-0.23024800	0.03353000
C	7.80008000	2.16759500	0.17444300
C	8.68223600	1.08061200	0.13736200
H	6.42211500	-1.46040800	-0.11253100
H	5.72788600	2.77268000	0.13583000
H	8.88912500	-1.06060100	0.00648300
H	8.18704000	3.18541800	0.25491800
O	10.01979600	1.22267700	0.19742900
H	10.24602300	2.15979100	0.26622000

Table S2. Cartesian coordinates (in Å) of the lowest energy (most stable) structure of AZO-Rhodamine2 in the NR state. The potential energy of the NR state (charge = +1, spin multiplicity = 1 [singlet]) is −46222.621147698 hartree, and that of the OER state (charge = 0, spin multiplicity = 2 [doublet]) is −46226.296237795 hartree. Computational level was ωB97XD/cc-pVDZ | IEFPCM(solvent = water).

Atom	x	y	z
O	0.96292700	-1.61943000	-0.02415200
O	2.01216300	4.06982100	2.69297700
O	1.56099700	1.93821400	2.17958700
N	5.53292700	-2.81092700	-0.00546600
N	-3.68969300	-0.96290900	0.02406300
C	1.55700900	1.09584400	-0.34802700
C	2.57213100	0.15742900	-0.28465500
C	0.19929600	0.65172200	-0.27006400
C	2.24719300	-1.22544800	-0.10665900
C	-0.04646300	-0.72230200	-0.10435600
C	4.57801600	-1.87567700	-0.09672600
C	-2.41604900	-0.35399800	-0.08705000
C	1.85683000	2.53181200	-0.61322300
C	3.20104600	-2.21071300	-0.01745700
C	-1.34031600	-1.22948000	-0.01189600
C	3.96774400	0.47746600	-0.36082300
C	-0.91645200	1.52074000	-0.34213700
C	4.92392900	-0.48026900	-0.27354300
C	-2.19817000	1.03440400	-0.25215400
C	5.21406000	-4.21761100	0.25446200
C	6.95946400	-2.50399500	-0.15684200
N	-4.65296700	-0.17061300	-0.11789800
C	1.98643300	3.48760300	0.40871000
C	2.00531000	2.92289000	-1.94559600
C	4.91939000	-4.99753900	-1.02152000
C	7.61336000	-2.09375200	1.15683900
C	2.26473200	4.81778300	0.07876000
C	2.27913500	4.25198300	-2.26298600
C	2.40911400	5.20064600	-1.25098600
C	1.82880400	3.06812800	1.83021900
H	2.85723900	-3.23488200	0.09661900
H	-1.50579200	-2.29813700	0.11851200
H	4.25889300	1.52057100	-0.48351500
H	-0.74538300	2.58989200	-0.46861400
H	5.96740700	-0.17995300	-0.32056800
H	-3.05144500	1.70708200	-0.30314200
H	6.08030500	-4.64837200	0.77444100
H	4.37632700	-4.27429400	0.96193100
H	7.43484000	-3.41102500	-0.55428800
H	7.09021200	-1.73460700	-0.92816900
H	1.90467800	2.17970900	-2.73786300
H	4.04311200	-4.58992000	-1.54661800
H	5.77829800	-4.96313700	-1.70793500
H	4.71675300	-6.04956500	-0.77558900
H	7.16385100	-1.17237500	1.55584900
H	7.50346000	-2.88476100	1.91341500
H	8.68639500	-1.91505500	0.99866700
H	2.36761700	5.55228300	0.87621200
H	2.39213200	4.54326000	-3.30794500
H	2.62470400	6.24087900	-1.49664200
H	1.88946200	3.70826300	3.58579000
C	-5.92669200	-0.71223900	-0.00704500
C	-6.97748200	0.21837200	-0.18473600
C	-6.23516300	-2.06090000	0.26146500
C	-8.30620200	-0.19773400	-0.09317000
C	-7.54368200	-2.47653600	0.35299100
C	-8.58481300	-1.53704800	0.17515700
H	-5.41276300	-2.76340600	0.39556000
H	-9.10581700	0.53148400	-0.23247800
H	-7.80073500	-3.51489400	0.56102900
O	-9.84010000	-2.00546000	0.27901100
H	-10.47220900	-1.28632500	0.14532200
O	-6.71779300	1.50640500	-0.44243500
H	-5.74803300	1.59254200	-0.46649600

Table S3. Cartesian coordinates (in Å) of the lowest energy (most stable) structure of AZO-Rhodamine3 in the NR state. The potential energy of the NR state (charge = +1, spin multiplicity = 1 [singlet]) is −49306.3085245246 hartree, and that of the OER state (charge = 0, spin multiplicity = 2 [doublet]) is −49310.0753422999 hartree. Computational level was ωB97XD/cc-pVDZ | IEFPCM(solvent = water).

Atom	x	y	z
O	1.367682000	-1.674115000	-0.074695000
O	1.792818000	4.172191000	2.453511000
O	1.597056000	1.987550000	2.006743000
N	5.968493000	-2.677746000	0.251838000
N	-3.300184000	-1.193793000	-0.386726000
C	1.879867000	1.051118000	-0.467732000
C	2.920704000	0.158585000	-0.303383000
C	0.533771000	0.552244000	-0.461414000
C	2.638402000	-1.231708000	-0.093614000
C	0.332889000	-0.822927000	-0.258820000
C	4.987418000	-1.785206000	0.077590000
C	-2.039100000	-0.548679000	-0.411193000
C	2.138208000	2.488122000	-0.766242000
C	3.620816000	-2.172562000	0.090225000
C	-0.942753000	-1.382994000	-0.245275000
C	4.306779000	0.532679000	-0.301347000
C	-0.602323000	1.369605000	-0.660364000
C	5.290626000	-0.381965000	-0.123857000
C	-1.869784000	0.835321000	-0.635320000
C	5.689248000	-4.087363000	0.543473000
C	7.389561000	-2.322572000	0.161077000
N	-4.255805000	-0.445893000	-0.097469000
C	2.092990000	3.492175000	0.216281000
C	2.419630000	2.831624000	-2.090239000
C	5.491052000	-4.919131000	-0.718204000
C	7.963872000	-1.858440000	1.493655000
C	2.330655000	4.822068000	-0.144081000
C	2.652101000	4.161022000	-2.438481000
C	2.607215000	5.157355000	-1.465889000
C	1.803559000	3.121877000	1.630638000
H	3.311025000	-3.205520000	0.221104000
H	-1.073904000	-2.453932000	-0.096722000
H	4.564283000	1.582591000	-0.439209000
H	-0.463365000	2.434958000	-0.843284000
H	6.322793000	-0.041776000	-0.114689000
H	-2.737124000	1.468755000	-0.813440000
H	6.543293000	-4.465774000	1.121010000
H	4.818847000	-4.153344000	1.209173000
H	7.914262000	-3.221353000	-0.190053000
H	7.529278000	-1.567721000	-0.622677000
H	2.453968000	2.051518000	-2.852004000
H	4.628972000	-4.563467000	-1.301291000
H	6.382975000	-4.875056000	-1.360460000
H	5.314606000	-5.969212000	-0.445522000
H	7.463877000	-0.944897000	1.847810000
H	7.845824000	-2.634985000	2.263785000
H	9.036327000	-1.645170000	1.381416000
H	2.298683000	5.593885000	0.623585000
H	2.870095000	4.414757000	-3.476577000
H	2.790104000	6.197860000	-1.735697000
H	1.597091000	3.839269000	3.344475000
C	-5.540828000	-1.024835000	-0.099328000
C	-6.628560000	-0.201467000	0.271814000
C	-5.770715000	-2.359273000	-0.461304000
C	-7.911502000	-0.734914000	0.267961000
C	-7.050803000	-2.882017000	-0.459004000
C	-8.133912000	-2.067472000	-0.093694000
H	-4.921054000	-2.978008000	-0.746921000
H	-8.752817000	-0.104179000	0.551346000
H	-7.218294000	-3.923097000	-0.742063000
O	-9.403257000	-2.506225000	-0.072226000
H	-9.438147000	-3.434030000	-0.341374000
C	-6.523344000	1.253576000	0.687167000
O	-7.511506000	1.903718000	0.947044000

Table 3 continued

O	-5.315363000	1.796607000	0.769082000
H	-4.643001000	1.119079000	0.502536000

References

[1] M. C. Sauer, S. Arai, L. M. Dorfman, *J. Chem. Phys,* **1965**, 42, 2, 708–712
[2] Frisch, M. J.; Trucks, G. W.; Schlegel, H. B.; Scuseria, G. E.; Robb, M. A.; Cheeseman, J. R.; Scalmani, G.; Barone, V.; Petersson, G. A.; Nakatsuji, H.; Li, X.; Caricato, M.; Marenich, A. V.; Bloino, J.; Janesko, B. G.; Gomperts, R.; Mennucci, B.; Hratchian, H. P.; Ortiz, J. V.; Izmaylov, A. F.; Sonnenberg, J. L.; Williams-Young, D.; Ding, F.; Lipparini, F.; Egidi, F.; Goings, J.; Peng, B.; Petrone, A.; Henderson, T.; Ranasinghe, D.; Zakrzewski, V. G.; Gao, J.; Rega, N.; Zheng, G.; Liang, W.; Hada, M.; Ehara, M.; Toyota, K.; Fukuda, R.; Hasegawa, J.; Ishida, M.; Nakajima, T.; Honda, Y.; Kitao, O.; Nakai, H.; Vreven, T.; Throssell, K.; Montgomery, J. A., Jr.; Peralta, J. E.; Ogliaro, F.; Bearpark, M. J.; Heyd, J. J.; Brothers, E. N.; Kudin, K. N.; Staroverov, V. N.; Keith, T. A.; Kobayashi, R.; Normand, J.; Raghavachari, K.; Rendell, A. P.; Burant, J. C.; Iyengar, S. S.; Tomasi, J.; Cossi, M.; Millam, J. M.; Klene, M.; Adamo, C.; Cammi, R.; Ochterski, J. W.; Martin, R. L.; Morokuma, K.; Farkas, O.; Foresman, *Gaussian 16 (Revision C.01)*, Gaussian, Inc., Wallingford CT, **2016**.
[3] Maeda, S.; Harabuchi, Y.; Takagi, M.; Saita, K.; Suzuki, K.; Ichino, T.; Sumiya, Y.; Sugiyama, K. & Ono, Y. *J. Comput. Chem.* **2018**, 39, 233–251.

Author Contributions

CRediT authorship contributions

Koki Ogawara: Investigation, Visualization, Writing, Data curation, Validation - original draft.

Dr. Hideo Takakura, Dr. Naoya Ieda: Investigation.

Dr. Kenichiro Saita, Dr. Sonu Kumar: Investigation, Data curation.

Dr. Kohei Nakajima: Investigation, Writing - review & editing.

Prof. Dr. Osamu Inanami, Prof. Dr. Tetsuya Taketsugu: Investigation, Resources.

Prof. Dr. Mikako Ogawa: Conceptualization, Project administration, Funding acquisition, Resources, Supervision, Writing - review & editing.
